# Multidimensional measure of instrumental support in transitional care – design and pilot test of a questionnaire assessing instrumental support among older adults with chronic diseases

**DOI:** 10.1186/s12877-022-03325-8

**Published:** 2022-08-01

**Authors:** Fengbo Yang, Jianing Hua, Guiling Geng, Min Cui, Wenwen Yang, Zihan Geng

**Affiliations:** 1 School of Medical, Nantong University, Nantong, China; 2 Affiliated Hospital of Jiangnan University, Wuxi, China

**Keywords:** Transitional care, Instrumental support, Older adult, Chronic disease, Questionnaire

## Abstract

**Background:**

Previous studies indicated that poor quantity and quality of instrumental support are one of the main barriers in the application of transitional care. Instrumental support, as one common function of social support, is the provision of financial assistance, material goods, or services. The purpose of our study is to develop an Instrumental Support in Transitional Care Questionnaire (ISTCQ) and use this questionnaire to make an assessment among older adults with chronic diseases.

**Methods:**

The draft questionnaire was examined by 18 experts from different professional fields performing three rounds of content validity testing with the Delphi method. Afterward, we conducted a pilot test recruiting 174 participants as a convenience sample in Nantong, China. The construct validity was confirmed via exploratory factor analysis and reliability was assessed using Cronbach's alpha.

**Results:**

The authority coefficient of experts was 0.74–0.99 and Kendall harmony coefficient W was 0.381. The exploratory factor analysis indicated that the questionnaire can be interpreted by three factors: namely, anticipated support (items 1, 2, 3, 4), received support (items 5, 6, 7, 8) and support satisfaction (items 9, 10, 11, 12). These three factors (eigenvalues > 1 and factor loading > 0.4) explained 69.128% of the total variance. Furthermore, the calculation of Cronbach's alpha and test–retest reliability have shown good reliability among each dimension of the 12-item questionnaire (Cronbach's alpha 0.711–0.827, test–retest reliability 0.704–0.818).

**Conclusion:**

Results from the pilot test demonstrated excellent reliability and validity of ISTCQ through each dimension and as an entire.

## Introduction

The aging population has grown into a concerning health issue worldwide, and this problem is accompanied by an epidemiological transition in which chronic diseases have overtaken infectious diseases as the world’s biggest killers [1]. The high prevalence of chronic diseases has brought great challenges, and to a certain extent, poses a huge financial and disease burden in many nations [2–4]. In particular, older adults with chronic diseases experience frequent changes in health status accompanied by multiple transitions within and among different healthcare settings [5]. This raises the risk of medication errors, treatment errors, and infections that can lead to unnecessary health expenditure and hospital readmission [6–8].

To address the negative effects of transitions among older adults with chronic diseases, innovative solutions aimed to improve integration and continuity across episodes of care have emerged. Collectively, these solutions are referred to as “transitional care” [9, 10]. Interventions of transitional care have been widely implemented in older adults with chronic diseases. These interventions have effectively lowered the rate of hospital readmissions and adverse events [11, 12]. However, some studies have pointed out that barriers remain in the delivery of transitional care, particularly given the unique conditions of each patient (e.g., socioeconomic status, caregiver support) [13, 14]. Moreover, care coordination problems are compounded by patient factors such as transportation or financial concerns, availability or accessibility of healthcare providers [15]. Even after receiving high-quality medical treatment, transitional care for geriatric patients can be hindered by a variety of systemic and social factors [16], such as limited coverage of medical insurance [17]. To sum up, key challenges in the implementation of transitional care interventions could be summarized as poor instrumental support, including a lack of funds, staff, and equipment [18].

Functions of social support are classified into different types in different studies. and the most commonly cited one is emotional support, tangible or instrumental support and informational support [19]. Instrumental support typically includes practical or tangible forms of support, such as funds, task assistance, and direct intervention on behalf of the recipient [18, 20]. Higher quantity and quality of instrumental support is linked to better health outcomes [21]. Hence, instrumental support is critical in transitional care for older adults with chronic diseases, due to the multi-sectoral coordinated efforts of transitional care. The needs for long-term healthcare coverage among older adults with chronic diseases also contribute to high demands for instrumental support. They need not only strong financial support, but also the support of healthcare professionals, family/friends, and fellow patients [22].

Despite the essential role of instrumental support in transitional care, few studies have examined its effects. This is because no uniform tools available for the measurement of instrumental support. The Care Transitions Measure (CTM) developed by Coleman [23, 24] and the questionnaire developed by Masters [25] assessed the quality of services in transitional care, but neither of them measured instrumental support. To fill the gaps, our study, for the first time, laid emphasis on the measure of instrumental support from the patients’ perspective and tested the reliability and validity of the tool—a questionnaire assessing instrumental support in transitional care.

## Methods

### Phase 1: ISTCQ Development

#### Literature review

We conducted a literature search related to the construct “instrumental support in transitional care for older adults with chronic diseases” using the Medline, PubMed, Web of Science, and CNKI databases. After screening the titles and abstracts of the literature, 12 pieces of literature were initially identified for further screening, and 6 pieces of literature were finally included to form the items pool by reading the full text of the literature [26–31].

#### Theoretical basis

Power et al. [32, 33] assessed the instrumental support according to the actual level of support and the expected ideal level of support, in order to elicit information regarding the function of social support for a range of key relationships in an individual's life, in both a realistic and idealistic sense.

#### Semi-structured interviews with experts

We developed guidelines for the semi-structured interview according to the components of instrumental support in transitional care and the existing measure of instrumental support in transitional care. A total of 16 managers from hospitals, nursing homes, community health centers and government staff underwent the semi-structured interview. Coding and analysis of the semi-structured interview data revealed that instrumental support in transitional care consists of service support, staff support, financial support, equipment and supplies support, which add the contents of items pool.

#### Item development

Items for the ISTCQ measure were developed deductively from theory as articulated in the above sections and inductively from qualitative interviews.

### Phase 2: Content validity testing

#### Sample

We used the Delphi anonymous consultation method to test the content of the questionnaire [34]. The preliminary version of the questionnaire consisted of 16 items, evaluated by an expert group (16 nursing specialists and 2 sociologists) with a higher education background (bachelor's degree and above). These experts worked in hospitals, nursing homes, and universities, and have considerable expertise in clinical nursing, nursing management, geriatric nursing, nursing education, community nursing and sociology.

One researcher emailed the experts with the designed anonymous consultation letters. All replies were collected within 2 weeks. Another researcher took charge of the data analysis collected from consultation letters to ensure the anonymity of the questionnaire. The experts were not aware of other participants. In total, three rounds of Delphi anonymous consultations were conducted, with a 90% response rate in the first round and a 100% response rate in the second and third rounds, which can be classified as good enthusiasm of experts.

#### Measure

After reaching a consensus, the final version of the Delphi expert consultation letter comprises: 1) Research purpose, significance and some explanations of how to fill the form; 2) Item importance assessment: A five-level Likert scale (where 1 = not important, 2 = somewhat important, 3 = neutral, 4 = quite important, 5 = highly important) [35] evaluating the degree of importance for each item in ISTCQ. The five-level Likert scale also contains columns for modifications, additions and deletions, 3) The expert information questionnaire includes basic information, such as age, education, working years, familiarity and judgment to the questionnaire. Familiarity can be divided into five levels (very familiar to very unfamiliar). The judgment includes four aspects: intuitive selection, practical experience, theoretical analysis and literature references.

#### Statistical analysis

The expert authority coefficient (Cr) reflects experts’ cognitive level of the research issues and is an important indicator of the reliability of the consulting results. The Cr is directly proportional to the expert's judgment coefficient (Ca) and familiarity coefficient (Cs) on each item. The formula for its calculation is: Cr = (Ca + Cs)/2, which is assessed by experts according to their actual situation. Generally, the Cr ≥ 0.7 indicates that the experts have high authority in this research field, and the questionnaire has certain credibility [36].

The concentrative degree of expert opinion is assessed by two indicators mean of item importance scores and coefficient of variation (CV). The CV reflects the fluctuation of experts' evaluation of item importance. It is generally believed that the smaller the coefficient of variation is, the more concentrated the expert opinions are, on the contrary, the more dispersed the expert opinions are. According to the item screening criteria, the items with mean < 4 and CV > 0.2 were excluded [37].

The coordinated degree of expert opinion is expressed by Kendall harmony coefficient (W). The value range of W is 0—1, where a higher value of W indicates a better coordination degree of experts' opinions.

### Phase 3: Cross-sectional validation survey

#### Sample

A convenience sample of patients was recruited from a general hospital and a community health center in Nantong, China to test the reliability and validity of the questionnaire. Patients were included if they met the following criteria: 1) at least 65 years old; 2) at least one diagnosed chronic disease (subject to the clinical diagnosis record); 3) received transitional care within the previous three months; 4) possession of reading and writing skills. The exclusion criterion was presenting a severe cognitive impairment (MMSE ≤ 9).

A group of three 3rd grade Master of Nursing students conducted a face-to-face questionnaire collection. A total of 180 questionnaires were distributed, 174 of which were returned with effective responses. All the participants provided verbal informed consent.

#### Measure

ISTCQ used in the pilot study was generated after Delphi anonymous consultation. ISTCQ consists of 2 parts: general information collection and the main body (ISTCQ Version 2.0). General information includes respondents’ gender, age, marital status, education level, monthly family income, medical payment method, diagnosis of major chronic diseases, monthly transitional care costs, and current status of social support. The main body of the questionnaire uses open questions except for the dimension of support satisfaction, which uses a five-level Likert scale.

#### Statistical analysis

The construct validity confirmed by the exploratory factor analysis (EFA), refers to the degree to which theoretical traits or concepts can be measured. Previously, we used the KMO and Bartlett test to test the suitability of the questionnaire for exploratory factor analysis. Factors were extracted based on eigenvalues of 1.00 or higher and factor loadings of greater than 0.40 [38]. If the correlation coefficient between two extracted common factors or the correlation coefficient between one certain common factor and the total score of the questionnaire was higher than 0.4, it indicates a good relevance [39].

The internal consistency is calculated to estimate the reliability of the questionnaire. It also assessed the consistency and stability of results from the questionnaire. The internal consistency is usually tested by Cronbach's alpha. Cronbach's alpha between 0.7 and 0.8 is acceptable, and 0.8 or higher is optimal [40]. Additionally, we selected 20 patients for repeated measurements to calculate the test–retest reliability after an interval of 2 weeks [41].

## Results

### Phase 1: ISTCQ Development (version 1.0)

According to the literature review and the theoretical basis, the following four dimensions emerged in ISTCQ (version 1.0): anticipated support, received support, subjective support, and support utilization. After the analysis of the semi-structured interviews conducted by the research team, we obtained themes related to instrumental support in transitional care, including service support, staff support, financial support, and equipment and supplies support. An initial battery of 16 items was generated.

### Phase 2: Content validity testing

#### Characteristics of consulting experts

The experts are 34—58 (46.53 ± 7.56) years old with working years of 6—37 (23.65 ± 9.39) years. Their education backgrounds are undergraduate or above, and their professional titles are sub-senior or above. The areas of expertise of these experts include clinical nursing, nursing management, geriatric nursing, nursing education, community nursing and sociology. Moreover, the expert authority coefficient (Cr) was calculated to be 0.74—0.99, greater than the cutoff value of 0.7. To sum up, the experts involved in Delphi anonymous consultation have extensive work and management experience, and highly professional and technical qualifications to ensure the reliability of the consulting results.

#### Revising the ISTCQ to version 2.0

Results of three Delphi rounds are shown in Table 1. After the first Delphi round, 10 of 16 items achieved a coefficient of variation (CV) less than 0.2. Through discussion, we deleted 6 items with CV greater than 0.2. Some experts suggested that the intensity of support is influenced by factors such as the supply side, the demand side and their satisfaction with the received support. Besides, subjective support and support utilization have a low fit towards our questionnaire. Therefore, we reached a consensus to replace these two dimensions (subjective support and support utilization) with support satisfaction. After the second Delphi round, 13 of 15 items achieved a CV less than 0.2. Some experts pointed out that it was difficult to accurately measure the actual circumstances of the participants through items of equipment and supplies support. After the discussion of the research group, A5, B5 and C5 were deleted. Other comments were primarily about the word using and options classification. After the third Delphi round, 12 items achieved a CV less than 0.2, with no item deleted. Although CV analysis showed excellent levels of consensus, the experts still suggested some minor revisions to certain items.

**Table 1:** Three round item and dimension importance scores

Round	Dimensions and items	‾*x*	*s*	*CV*
**1**	**A Anticipated Support**	4.53	0.606	0.13
	A1 Anticipated service support	4.71	0.456	0.10
	A2 Anticipated ways to receive service support	4.47	0.776	0.17
	A3 Anticipated staff support	4.53	0.606	0.13
	A4 Anticipated financial support	4.59	0.492	0.11
	A5 Anticipated equipment and supplies support	4.29	0.824	0.19
	**B Received Support**	4.88	0.322	0.07
	B1 Received service support	4.71	0.456	0.10
	B2 Ways to receive service support	4.88	0.322	0.07
	B3 Received staff support	4.65	0.588	0.13
	B4 Received financial support	4.47	0.606	0.14
	B5 Received equipment and supplies support	4.35	0.836	0.19
	**C Subjective support**	4.29	0.824	0.19
	C1 Subjective application of service support	4.24	0.876	0.21
	C2 Subjective application of staff support	4.53	0.977	0.22
	C3 Subjective application of financial support	4.00	1.138	0.28
	C4 Subjective application of equipment and supplies support	4.06	1.056	0.26
	**D Utilization of support**	4.76	0.941	0.20
	D1 Utilization of staff support	4.53	0.977	0.22
	D2 Utilization of financial support	4.12	1.131	0.27
**2**	**A Anticipated Support**	4.59	0.492	0.11
	A1 Anticipated service support	4.88	0.322	0.07
	A2 Anticipated ways to receive service support	4.82	0.381	0.08
	A3 Anticipated staff support	4.82	0.381	0.08
	A4 Anticipated financial support	4.65	0.588	0.13
	A5 Anticipated equipment and supplies support	4.76	0.941	0.20
	**B Received Support**	4.94	0.235	0.05
	B1 Received service support	4.88	0.322	0.07
	B2 Ways to receive service support	4.82	0.513	0.11
	B3 Received staff support	4.82	0.513	0.11
	B4 Received financial support	4.82	0.513	0.11
	B5 Received equipment and supplies support	4.53	0.606	0.13
	**C Support Satisfaction**	4.82	0.381	0.08
	C1 Service support satisfaction	4.88	0.322	0.07
	C2 Satisfaction of ways to receive service support	4.76	0.424	0.09
	C3 Staff support satisfaction	4.59	0.600	0.13
	C4 Financial support satisfaction	4.53	0.696	0.15
	C5 Equipment and supplies support satisfaction	4.53	0.977	0.22
**3**	**A Anticipated Support**	4.94	0.235	0.05
	A1 Anticipated service support	5.00	0.000	0.00
	A2 Anticipated ways to receive service support	4.76	0.424	0.09
	A3 Anticipated staff support	4.82	0.381	0.08
	A4 Anticipated financial support	4.76	0.424	0.09
	**B Received Support**	4.94	0.235	0.05
	B1 Received service support	4.88	0.322	0.07
	B2 Ways to receive service support	4.94	0.235	0.05
	B3 Received staff support	4.94	0.235	0.05
	B4 Received financial support	4.88	0.322	0.07
	**C Support Satisfaction**	4.76	0.424	0.09
	C1 Service support satisfaction	4.88	0.322	0.07
	C2 Satisfaction of ways to receive service support	4.82	0.381	0.08
	C3 Staff support satisfaction	4.82	0.381	0.08
	C4 Financial support satisfaction	4.82	0.513	0.11

*CV* Coefficient of Variation

Items with coefficient of variation higher than 0.20 were deleted

The Kendall harmony coefficient showed that W value of the third Delphi round was significantly higher than the first round and the second round, and the significance test results were *p* < 0.05 (Table 2). Results indicated that expert opinions and consultation results on the project showing high consistency are desirable.

**Table 2:** Kendall W value and chi-square test results

Round	Variables	N	*W*	*χ*^*2*^	*df*	*p*
**1**	Dimensions	4	0.128	4.343	2	0.014
	Items	16	0.165	53.134	19	0.007
**2**	Dimensions	3	0.190	14.797	3	0.002
	Items	15	0.267	72.670	15	0.000
**3**	Dimensions	3	0.359	19.540	2	0.000
	Items	12	0.381	89.176	14	0.000

According to the results and suggestions from three Delphi rounds, the ISTCQ (version 2.0) contains three dimensions of anticipated support, received support and support satisfaction and 12 items (details in Table 3).

**Table 3:** Compositions of the Instrumental Support in Transitional Care Questionnaire (Version 2.0)

Dimensions	Items
A. Anticipated Support	A1. What kind of services do you expect to receive in transitional care?
	A2. In what ways do you expect to receive services in transitional care?
	A3. Who do you expect to provide services in transitional care for you?
	A4. What kind of financial support do you expect to receive in transitional care?
B. Received Support	B1. What kind of services did you receive in transitional care?
	B2. In what ways did you receive services in transitional care?
	B3. Who provided services in transitional care for you?
	B4. What kind of financial support you received in transitional care?
C. Support Satisfaction	C1. Are you satisfied with the services you received in transitional care?
	C2. Are you satisfied with the ways to receive services in transitional care?
	C3. Are you satisfied with the people who provided services in transitional care for you?
	C4. Are you satisfied with the financial support you received in transitional care?

### Phase 3: Cross-sectional validation survey

#### Demographic and clinical characteristics of the participants

The demographic and clinical characteristics of the subjects who participated in the survey are shown in Table 4.

**Table 4:** Demographic and clinical characteristics of the participants (*n* = 174)

Item		Number	Percentage(%)
Age	60 ~ 69	53	30.5
	70 ~ 79	61	35.1
	≥ 80	60	34.4
Gender	Male	82	47.1
	Female	92	52.9
Marital status	Married	157	90.2
	Single	17	9.8
Level of education	Primary school or below	57	32.8
	Junior middle school	58	33.3
	High school	36	20.7
	College and above	23	13.2
Past occupation	Unemployment	10	5.7
	Farmer	22	12.6
	Worker	80	46.1
	Enterprise employee	52	29.9
	Public servant	10	5.7
Close friends or relatives	1 ~ 2	15	8.6
	3 ~ 5	71	40.8
	> 5	88	50.6
Main caregivers	None	27	15.5
	Partner	77	44.3
	Children	62	35.6
	Family nanny	6	3.4
	Relative	2	1.2
Household monthly income (yuan/month)	< 2000	33	18.9
	2000 ~ 4000	74	42.5
	4001 ~ 6000	25	14.4
	6001 ~ 8000	13	7.5
	8001 ~ 10,000	13	7.5
	> 10,000	16	9.2
Mode of medical expense	Urban resident's insurance	19	10.9
	Urban employee insurance	123	70.6
	Rural cooperative medical insurance	25	14.4
	Other	7	4.1
Number of chronic diseases	1	62	35.6
	2	69	39.7
	≥ 3	43	24.7
Transitional care expense (yuan/month)	< 500	88	50.6
	500 ~ 1000	48	27.6
	> 1000	38	21.8

#### Construct validity

**Fig. 1:**
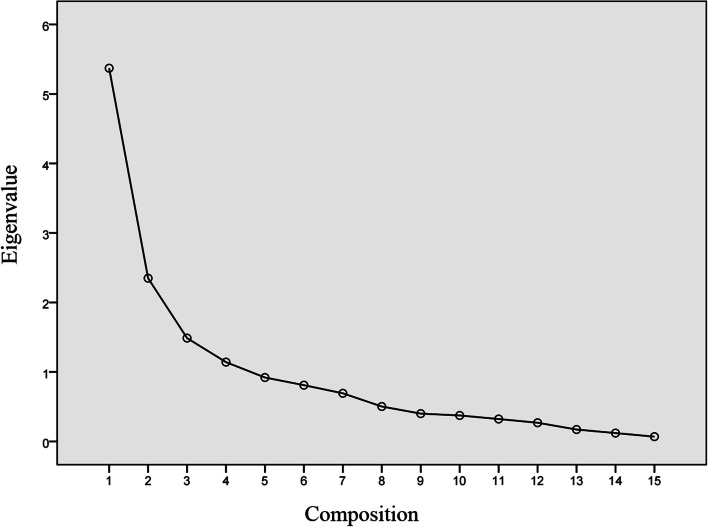
The scree plot

A total of 174 participants completed the ISTCQ and the data collected was used for factor analysis. This sample is a sufficient size for EFA [42]. KMO was 0.830 and the Bartlett sphericity test was < 0.05 (chi-squared, 904.764; degrees of freedom, 66). These indicate that ISTCQ was suitable for factor analysis [43]. We used the principal component analysis and varimax orthogonal rotation for exploratory factor analysis. EFA of the 12 items produced a three-factor solution based on the common factor eigenvalue greater than 1 (Fig. 1). The first factor created was summarized as “anticipated support”, consisting of four elements. The second factor is called “received support” with four elements. The last factor is “support satisfaction” including four elements (shown in Table 5). All of the items had higher item loadings on the corresponding common factors, ranging from 0.483 to 0.885. At the same time, the combined interpretation variance of the three factors was 69.128%, higher than the minimum criteria of the sum of eigenvalues (50%) [44]. Moreover, the correlation coefficients between each factor were from 0.592 to 0.785 (*p* < 0.01) (Table 6).

**Table 5:** Factor loadings of the ISTCQ items

Items	Factor			Commonality
	**Anticipated support**	**Received support**	**Support satisfaction**	
A1	0.868	-	-	0.846
A3	0.811	-	-	0.783
A2	0.691	-	-	0.618
A4	0.666	-	-	0.675
B1	-	0.885	-	0.800
B2	-	0.834	-	0.745
B4	-	0.691	-	0.582
B3	-	0.632	-	0.659
C2	-	-	0.654	0.650
C4	-	-	0.611	0.598
C3	-	-	0.552	0.456
C1	-	-	0.483	0.488
Eigenvalue	4.756	1.488	1.212	
Cumulative explained variance (%)	39.633	52.030	69.128	

*ISTCQ* Instrumental Support in Transitional Care Questionnaire

**Table 6:** Correlations between each factor (*r* value)

	**Anticipated support**	**Received support**	**Support satisfaction**
Anticipated support	1	—	—
Received support	0.785^**^	1	—
Support satisfaction	0.592^**^	0.625^**^	1
Questionnaire overall	0.931^**^	0.920^**^	0.742 ^**^

^*^^*^ *p* < 0.01

#### Internal consistency

The Cronbach’s α was 0.827, which indicated a good internal consistency. Cronbach’s α of the first factor “anticipated support” was 0.743; Cronbach’s α of “received support” was 0.711 and Cronbach’s α of “support satisfaction” was 0.764. The test–retest reliability was good, with Cronbach’s α of 0.818 (Table 7). The test–retest correlation in the present study was 0.985 at the group level (*p* < 0.001) and the confidence interval of the correlation was narrow (95%CI = 0.978–0.990).

**Table 7:** Internal consistency test results of ISTCQ

	**Cronbach's α**	**Test–retest reliability (*n* = 20)**
Anticipated support	0.743	0.736
Received support	0.711	0.704
Support satisfaction	0.764	0.753
Questionnaire overall	0.827	0.818

*ISTCQ* Instrumental Support in Transitional Care Questionnaire

## Discussion

We developed ISTCQ—a questionnaire measuring instrumental support in transitional care. A pilot test was followed among older adults with chronic diseases to test the reliability and validity of ISTCQ. The study was conducted in three phases, ISTCQ development, ISTCQ validation, and a cross-sectional validation survey. Each phase follows the recommendations for questionnaire development [45].

Results from ISTCQ development were a basis for the concept of ISTCQ and item development. Interviews with experts underscored the importance of instrumental support in transitional care. According to the interviews, we concluded the four dimensions of instrumental support in transitional care: service support, staff support, financial support, and equipment and supplies support. Hence, the preliminary formation of ISTCQ included 4 dimensions (anticipated support, received support, subjective support, and support utilization) and 16 items.

In ISTCQ validation, two dimensions subjective support, and support utilization were replaced with support satisfaction after the first round of Delphi expert anonymous consultation. Three items were deleted after the second round, and appropriate adjustments and modifications were made to certain items in the last round. The experts who participated in three rounds specialized in clinical nursing, nursing management, geriatric nursing, nursing education, community nursing and sociology. Their authority coefficients ranged from 0.74 to 0.99, showing a high degree of credibility. More importantly, the Kendall W value was higher in the third round than in the first and second rounds, and the CV was lower in the third round than in the first and second rounds; thus, there was a convergence of expert opinions. After modification and screening of questionnaire items through Delphi method, the final version of ISTCQ contains 3 dimensions (anticipated support, received support, support satisfaction) and 12 items.

A cross-sectional validation survey was conducted in the last stage as a pilot study. During the test, we found grammatical problems in some items and adjusted them immediately. The EFA findings indicated that the ISTCQ can be interpreted by three factors: namely, anticipated support (items 1, 2, 3, 4), received support (items 5, 6, 7, 8) and support satisfaction (items 9, 10, 11, 12). Cronbach’s alpha for the ISTCQ (0.827) showed that all items had high consistency, demonstrating satisfactory reliability of internal consistency. The results are similar to previous studies on measuring the informational support of older adults with chronic diseases in transitional care [46]. In practice, few studies measured instrumental support alone. Instrumental support is usually included social support as a certain dimension of social support [47, 48]. However, instrument support alone is complex and the measurement of instrumental support divers among different contexts in different studies. Therefore, our study hopes to develop an effective measure of instrumental support in transitional care.

According to the content of ISTCQ, we can assess the anticipated and received instrumental support in transitional care for older adults with chronic diseases. Differences in these two dimensions are an indicator of the gaps between demands among elderly chronically ill patients and the current situation of support. Through comparison, we can ascertain other instrumental support that is not anticipated and received in transitional care. Based on that, the transitional care team can provide targeted services, staff and funds to reduce unnecessary waste of resources. In terms of the support satisfaction dimension, we can see the level of satisfaction with the service support, staff support, and financial support in transitional care. The sum of scores from received support and support satisfaction could be calculated to show the quality of instrumental support. The practical application of our questionnaire in clinical work needs further study, and we will continue to report the follow-up results.

### Limitations

First, a known limitation of satisfaction measurement is a positively skewed distribution of scores in which respondents tend to rate items more favorably than unfavorably. Second, ISTCQ is the first questionnaire to investigate the instrumental support in transitional care and applied to older adults with chronic diseases. The practical application of ISTCQ needs further testing and cultural debugging in the future. Third, to meet the demand of the study, we will exclude patients with severe cognitive impairment. However, these excluded patients typically normally need more instrumental support than other patients.

## Conclusions

This study revealed that instrumental support in transitional care is a multidimensional construct that can be measured by 12 items in 3 dimensions (anticipated support, received support and support satisfaction). Qualitative and quantitative data were collected and analyzed to determine and validate ISTCQ. The ISTCQ is a valid measure with good reliability and validity. This questionnaire is recommended to be used to measure the instrumental support such as services, staff and funds in transitional care.

## Data Availability

The data that support the findings of this study are available from the corresponding author upon reasonable request.
